# Student and Nature Interactions and Their Impact on Mental Health during the COVID-19 Pandemic

**DOI:** 10.3390/ijerph19095030

**Published:** 2022-04-21

**Authors:** Jonah E. Trevino, Muntazar Monsur, Carol S. Lindquist, Catherine R. Simpson

**Affiliations:** 1 Department of Plant and Soil Sciences, Texas Tech University, Lubbock, TX 79409, USA; jonah.trevino@ttu.edu; 2 Department of Landscape Architecture, Texas Tech University, Lubbock, TX 79409, USA; mmonsur@ttu.edu; 3 Department of Sociology, Anthropology, and Social Work, Texas Tech University, Lubbock, TX 79409, USA; carol.lindquist@ttu.edu

**Keywords:** university students, mental health, COVID-19 pandemic, nature interactions, passive impacts, plants

## Abstract

Passive and active interactions with nature reduce stress, anxiety, and depression. Populations that experience increased stress often have fewer interactions with nature due to many factors. More recently, the COVID-19 pandemic has created a new stressor for all populations due to sickness, isolation, financial burdens, or other factors. University students were particularly impacted due to the change to online modalities, which isolated them from other students. To assess if any negative or other consequences were experienced and if nature factors could mitigate them, we examined how plant interactions affected university students (N = 353) in an online learning environment. Two modified Depression Anxiety Stress Surveys (DASS; Depression Anxiety Stress and Academic Stress, DASA) were administered over two semesters in 2020 to survey students on these interactions with nature. During the two semesters, most students experienced extremely severe self-reported mental health adversities. Further correlations between DASA scores and responses about nature interactions, home environments, plant exposure, and plant access showed that outdoor interactions were positively related to better self-reported mental health scores. However, the concerning and lingering effects of the pandemic were evidenced in our research as DASA scores increased across the two semesters. Nevertheless, going outdoors and interacting with nature brings some benefits that lessen the severity of depression, anxiety, and stress.

## 1. Introduction

The COVID-19 pandemic has profoundly altered students’ lives at all grade and developmental levels. During normal years, university students are subject to many stressful conditions that are highly influenced by their environment, peers, and academic disciplines. These stressful factors were then exacerbated by the pandemic. During the height of the pandemic, students faced the rigors of university education in an online learning environment, which enhanced stress on many levels. This circumstance ultimately changed how students experienced the college lifestyle and introduced new stressors to the academic setting, some of which are still present to date. Along with changes in social activities such as ceasing students’ recreational activities on campus, negative emotions due to the online shift have also been found [1,2]. In addition to pressure associated with class performance, students were burdened with problems such as financial insecurity, uncertainties in romantic relationships, health, family, death, and their own isolation from peers and loved ones [3,4,5]. Literature published within the last two years has shown that the COVID-19 pandemic has resulted in increased psychological stress in students [6]. Furthermore, university student-athletes, and females, have been increasingly affected by depression, anxiety, and stress [2]. This has been a common theme worldwide, and a study of online learners in China found that depression, anxiety, and stress were high, specifically among males and those studying subjects other than medicine [1]. This snapshot of literature attests to the wide-spread impact of COVID-19, but the question remains on what factors or resources may counteract or ameliorate the stressors and negative consequences associated with a pandemic. Furthermore, how do nature or natural elements (e.g., greenspaces, window views, and interior plants) affect online learners at an American university?

Natural environments provide space and distance from other people while still allowing interaction with fewer safety risks [7,8]. However, few studies have had the opportunity to explore the impacts of nature on students in online learning environments, and furthermore, how the experience of a nationwide pandemic affected students with regard to depression, anxiety, stress, and academic stress. Outside of the pandemic, previous studies have shown that natural environments can affect student performance, mental health, and satisfaction with their academic courses [9,10]. These studies found that that window views, campus greenspaces, and passive and active interactions with plants can have overall positive effects on students [11,12,13]. Interactions with these nature-based elements are therefore potential mechanisms to reduce mental strains and improve emotional well-being. Most university campuses have greenspaces, and many college students frequently use these greenspaces for personal enjoyment [14]. In one study, students deemed outdoor spaces with more greenery to be more likely to have mentally restorative effects [15]. Overall, outdoor greenspaces and walks through natural settings on college campuses can improve the quality of life among those who utilize these areas [13]. Window views of nature also play a role in people’s mental state. When university students have the option of studying with plants or natural window views versus rooms without plants or windows with hardscape views, they are often drawn to areas with natural elements [11]. Benfield et al. [16] researched students’ exposure to window views with nature versus concrete walls and showed that students who were exposed to natural views had a higher level of satisfaction with their courses and higher end-of-semester grades. Moreover, a researcher interviewed students who were exposed to plants in a classroom environment and found that these items boosted social comfort and enhanced collaboration [17]. Interior plants in a classroom have also been shown to increase student participation in lectures [9]. These results provide evidence that greenspaces can greatly influence a student’s daily life in a university setting.

However, mental health and well-being are subjective factors that can be difficult to measure [18]. Therefore, tools have been developed to measure factors that detract from positive mental health, such as depression, anxiety, and stress [19]. Among these tools is the Depression, Anxiety, and Stress Scale (DASS) [20] which has been used frequently to assess mental health on many populations and has been a proven tool to measure student populations [2,6]. Depression, anxiety, and stress are common in populations throughout the world and are triggered by different individual experiences [21]. They are interrelated and can have common symptoms and degrees to which they are expressed [22]. Depression is characterized by negative feelings, sadness, low self-esteem, and loss of interest which can affect appetite, sleep, energy levels, and cognitive function [23]. Anxiety differs in that feelings of anxiety and fear are more predominant than those related to sadness [23]. Stress is more difficult to define in that it can be associated with an environment or environmental factors (e.g., cold or heat), be a response to external pressures, or a psychological response [24]. Academic-induced stress is stress experienced in association with or because of pressures related to academic activities [25]. Academic-induced stress is common in students who are enrolled in a college or university, and the causes can range from the cost of tuition to interpersonal relationships or the pressures of demanding programs [26]. Once students are in a university setting, academic performance-related stress becomes one of their more common stress factors, with 55% of students in the U.S. reporting this claim [27]. Students may also experience difficulties in their interpersonal relations from the strains associated with going through a life-stage change, adjusting to a new living environment, and working to fulfil academic requirements [28,29]. A recognized stressor is the financial burden associated with attendance [30]. Due to these stressors, students may seek mechanisms to alleviate the stress, such as experiencing nature by going outside, or negative escapes such as drugs or alcohol. 

Nature has long been a source of restoration and refuge for those who are experiencing hardships. This is also true for populations suffering from the COVID pandemic, who used this opportunity to go outside and experience nature in various ways. Buckley and Westaway [7] hypothesized that outdoor tourism would be essential for recovery from COVID, especially for urban women with families. This claim coincided with another study that theorized that using nature to heal the mental anguish that was induced by COVID could be extended to all demographics [31]. Furthermore, it was noted that people were going out into nature more often during the pandemic. Morse et al. [32] saw that Vermont residents were spending more time in nature and that this heightened relationship was extremely important during the first few months of the pandemic. Local state parks and natural recreational areas also had an increase in the number of visitations during 2020, when compared to 2019 [8,33]. Additionally, sales of outdoor gear increased, and forest therapy became increasingly popular [8]. To date, there is little to no literature about how students in in-home learning environments interacted with nature or plants. Therefore, we hypothesized that interacting with nature or plants would positively affect students that were subject to at-home orders during the COVID-19 pandemic. The objectives of this study were to assess students’ stress in home-learning environments and determine if plant interactions played any role in reducing their stress.

## 2. Materials and Methods

### 2.1. Survey Topics

Both surveys had the same questions for all participants to answer; however, some questions had additional answer choices for the Fall 2020 survey for students who participated in in-person classes. The additional answer choices were adjusted for students who studied on-campus. For example, if a question asked, “Where do you spend time working?”, the Summer 2020 survey listed areas that were in their own home environment, while the Fall 2020 survey listed “on-campus” as an option. The survey contained multiple sections to evaluate demographics, mental health, attitudes towards COVID-19, work/study environments, and interactions with natural elements. The DASS survey section was followed by academic stress questions, workplace environment questions, COVID-19 stress questions, and nature exposure questions. 

#### 2.1.1. DASS Survey

The survey used in this study was based on the Depression Anxiety Stress Scale (DASS), which was slightly modified for this project [20]. To the original range of responses, we added a fifth option: “Applies all the time.” This extra answer was added to adjust for the high-stress situation many students were facing during the COVID-19 pandemic. The first 23 questions of this current survey were taken directly from the DASS; however, two questions were omitted from analysis to match questions listed in the DASS 21 for analysis (Table A1). 

#### 2.1.2. Academic Stress Questions

The remaining questions, written specifically for the DASS survey section, were designed to focus on stressors induced by academia and the academic environment. This section is referred to as “academic stress.” To distinguish it from the original survey, this study’s modified DASS survey is referred to as the DASA (Depression, Anxiety, Stress, Academic Stress) survey throughout the remainder of this article (Table A1).

#### 2.1.3. Workplace Information Survey Questions

Questions regarding the “at-home” environment assessed students’ COVID-19 learning environment by asking about workplace set-up (referred to as ‘workplace functionality’) (Table A2) during at-home learning and any limitations (referred to as ‘workplace constraints’) (Table A3) that an individual may have experienced. Researchers also asked if there were any difficulties getting online (Table A4). Workplace functionality had participants select which environmental factors were present at their workplace, and workplace constraints listed different factors that may have made working at home difficult. 

#### 2.1.4. COVID-19 Stressor Survey Questions

The COVID-19 Stressor survey questions were located within the section dedicated to asking students not only about their experiences working at home, but also how COVID-19 affected them on a personal level. Questions related to the effects COVID-19 had on finances (Table A5), student experiences (Table A7), if any positive effects were experienced (Table A8), etc., were asked to determine if any direct consequences of the pandemic were experienced that were not covered in other sections. These were taken into consideration due to the mental struggle that may, or may not have, affected students during the COVID-19 pandemic

#### 2.1.5. Exposure to Nature Survey Questions

Another section assessed the “Individual’s Exposure to Nature”. This section asked 12 questions which evaluated students’ active and passive interactions with nature on a daily basis. This section also contained questions on hobbies, jobs, or regular interactions with plants. Researchers sought to record participants’ plant access (Table A6) during their hours working from home, indoor plant exposure they experienced at home (Table A9), and their amount of time experiencing nature in the outdoors (Table A10). This section assisted in printing a snapshot of a student’s daily plant exposure over the course of the initial stay-at-home order during the pandemic. 

### 2.2. Participants

This survey assessed depression, anxiety, stress, and academic stress of university students who engaged in online learning during the COVID-19 outbreak, and the impact of nature-based associations on these variables. The first part of the survey collected information on respondents’ basic demographics: age, student status, gender, and major. Any student who was currently enrolled in Texas Tech and over the age of 18 was eligible to participate. This guaranteed that only students would be allowed to participate in the survey, ensuring that the target population was achieved. An Institutional Review Board (IRB) review was conducted prior to administering the surveys and permission was granted to perform this research.

### 2.3. Survey Distribution

Students at Texas Tech University were sent “TechAnnounce” messages about this survey at regular intervals throughout the summer and fall semesters of 2020. Out of the 40,322 students enrolled during the 2020 school year, we had a total of 353 respondents, making the response rate 0.9%. Texas Tech’s “TechAnnounce” is a daily email that contains university-wide notifications and information. In accordance with their recruitment periods, the responses to these surveys were grouped into “Spring/Summer 2020” and “Fall 2020” categories. Survey Monkey (Momentive, San Mateo, CA, USA) was used to distribute surveys to students.

### 2.4. Data Analysis and Reliability 

Data were prepared by coding similar answers with a uniform designator so that a mixed-methods analysis could be performed. Timeline, data collected, number of responses, timeframe of data collection, user groups, population, and distribution were compared using analysis of variance where appropriate. Where DASA scores were collected, questions were separated by category and correlated to DASA scores using fit models in JMP 15.0.0 (SAS, Cary, NC, USA). A Cronbach’s alpha test was performed on data obtained from DASA questions, showing a reliability of >0.9 for depression, anxiety, and stress sections and >0.8 for the academic stress section, indicating suitable reliability for the overall instrument [34]. The DASA questions were the same for both Spring/Summer and Fall semesters. Since the surveys differed slightly in the Spring/Summer and Fall 2020 semesters, they were not compared directly during analysis. Survey questions that were used for correlations had a list of answers for participants to select. A cumulative score was derived from summing the number of answers selected for each categorical question that can be found in the Appendix A. Those scores were then correlated to DASA scores using multivariate (Spearman’s ρ) correlation analysis.

## 3. Results

### 3.1. Demographics

The demographics for the student populations are shown in Table 1. The sample size for Spring/Summer and Fall 2020 were 159 and 194, respectively. Most students fell within the 18–24-year-old age range and graduate students represented the largest proportion of respondents. Gender distribution was skewed to a female-student majority and most students did not have an academic major or minor that involved plants or plant sciences. Finally, prior to the issuance of COVID-19-related off-campus learning orders, almost all of the participants were on-campus students.

### 3.2. DASA Scores

For the depression, anxiety, and stress scores, during both semester categories, most of the participants scored “extremely severe,” the highest score possible. There was a bimodal, almost multi-modal nature to responses for the DASS scores. The second most-frequent score ranking was “normal,” the lowest score possible. For the academic stress scores, Spring/Summer semester respondents had mostly normal and mild scores, while Fall semester respondents had mostly mild and moderate scores (Table 2).

### 3.3. Anxiety and Stress Scales by Gender

Participants’ gender had significant effects on anxiety and stress scores. Female participants on average reported moderate to severe anxiety scores during the Spring/Summer semester, and moderate to severe stress scores during both Spring/Summer and Fall (Table 3). Comparatively, male respondents reported lower scores for anxiety and stress.

### 3.4. Response to Nature

When looking at responses to the question, “If you do go outside, how do you feel when you return home?”, time outside correlated with significant effects on all depression, anxiety, stress, and academic stress scores in both semesters. Participants who reported feeling an enhancement of focus when they returned home after being outdoors had significantly lower DASA scores compared to those who felt worse when returning home, and/or did not go outside at all (Table 4).

### 3.5. Correlation between DASA Scores and Responses to Nature

For the Spring/Summer semester, there were several significant correlations between measured survey responses. Indoor plant exposure was positively correlated with outdoor exposure, school–workplace constraints, and plant access (Table 5). Notably, outdoor exposure was negatively correlated with COVID-19 stress and DASA stress scores, and positively correlated with plant access and indoor plants. Furthermore, COVID-19-related stress was negatively related to outdoor exposure and beneficial effects perceived by respondents, but it was positively related to workplace constraints, difficulties getting online, financial issues, and DASA scores. This further illustrates not only the impacts of COVID-19 stress on students but also that some of this stress could possibly have been overcome by outdoor exposure (Table 5). Some notable trends worth mentioning were that outdoor plant exposure seems to be negatively correlated with DASA scores and COVID-19-related stressors. Furthermore, students with a highly functional workplace and plant access seemed to also have lower DASA scores (Table 5). 

During the Fall semester, many findings were similar to those in the Spring/Summer survey. Respondents who reported higher indoor plant exposure also reported higher outdoor exposure, workplace functionality, and plant access (Table 6). Higher outdoor exposure ratings were significantly related to lower depression, stress, and academic stress scores in participants. If participants deemed COVID-19 to be beneficial for their academic experiences, then their depression, anxiety, stress, and academic scores were lower. Furthermore, students that saw the beneficial aspects of COVID-19 also had higher outdoor exposure, workplace functionality, plant access, and lower reported COVID-related stress scores. Another noticeable trend that correlations expressed was that indoor plant exposure, outdoor plant exposure, and workplace functionality were negatively correlated with DASA scores (Table 6). 

## 4. Discussion

During the Fall semester, a significant upward trend was found for depression, anxiety, stress, and academic stress compared to the Summer semester. Early research published in March 2020 anticipated that many mental health issues would arise during the months following the initial quarantine and shelter-in-place orders, and that they would worsen as these restrictions continued [35,36]. This evidence illustrates the severity of the COVID-19 outbreak on student stress and overall mental health. Many students were aware of the impact that COVID-19 had had on their lives, whether it was through being at home, impacts on relationships, or an increased amount of academic stress. Recently published literature regarding the COVID-19 pandemic also found signs of deteriorating mental health in the general public [37,38]. We show that, in the college-student demographic, the pandemic had profound effects on mental and emotional health, both of which declined as the pandemic restrictions progressed.

During the Fall semester, students’ depression scores significantly decreased when they started spending more time outdoors, and they recognized an improvement in mood. When asked, “Do you find yourself spending more time outdoors due to the current outbreak? If you are, do you think this is affecting your mood/ stress?”, students who responded positively to both questions had significantly lower depression scores in comparison to those who were not going outside. Students who felt worse when they went home after being outside or were not going outside at all had notably higher DASA scores in both semesters when compared to those who felt “an enhancement of focus” when returning home. This extended benefit of outdoor exposure is promising, particularly in regard to maintenance of mental health over a long period of time. Students who reported feeling “very good” after returning from nature had significantly lower DASA scores compared to those who felt worse or did not go outside, but that improvement was not as profound as it was among students who experienced restorative effects when returning home. These results showed that a person’s attitude and their awareness of noticeable differences in mood, in addition to how environments may influence both factors, are extremely important in realizing benefits from nature encounters. These findings significantly reflect Stephen Kaplan’s [39] research focusing on nature’s role in Attention Restoration Theory, which shows how the restorative effects of nature can play a role in decreasing stress and improving mental health. The research conducted in this study, together with the recently published literature regarding people’s involvement with nature during COVID-19, demonstrates that people recognize the benefits of being outside and are actually going outdoors more frequently, which underscores the importance of exposure to nature in their mental wellbeing. 

A multivariate correlation analysis of both semesters’ question categories along with DASA scores further illustrated the impact and relationships between DASA scores and student stress factors that were related to COVID-19 issues. During the Spring/Summer semester, outdoor exposure significantly reduced COVID-19-related stress, but the relationship was not significant during the Fall semester. This difference may have been due to the likelihood that outdoor activities increased during the Spring/Summer, with the accompanying effects of exposure to nature that alleviated stress [40,41]. Outdoor exposure did significantly reduce stress scores during the Spring/Summer while specifically reducing depression, stress, and academic stress during the Fall. These results show that nature still had a beneficial effect on student mental health; however, it was not enough to entirely mitigate all factors related to pandemic-induced stressors. 

Interestingly, when students had a positive attitude toward COVID-19 compared to being consciously, negatively affected by the pandemic, they also reported lower depression, anxiety, stress, and academic stress. Yet, those who reported more beneficial effects of the pandemic also reported higher outdoor exposure and workplace functionality. These results indicate that attitudes towards COVID-19 may not be straightforward but may be influenced by environmental or socioeconomic advantages. Conversely, those who reported more COVID-19-related stress also reported more workplace constraints, difficulties getting online, financial issues, and worse mental health. This also implies that accessibility to outdoors, a good workplace, and adequate internet may disproportionally affect those without financial means. While students who reported higher COVID-19-related stress in the Fall semester did not significantly benefit from outdoor exposure, this was most likely due to the chronic stress from the pandemic [42]. This finding coincides with other research, as those who experience high-stress situations cannot gain significant relief in coping with their circumstances from just nature alone, although the natural interactions do assist in improving quality of life [43]. While these results indicate that the best method to reduce COVID-19-related stress was by keeping a positive outlook on the situation, that might not have been possible for everyone, particularly those with more financial and environmental constraints. However, it is clear that plant interactions did play a significant role in alleviating some of the mental taxation, stress, and other negative factors that were associated with the worldwide pandemic.

### Limitations

This research sought to investigate how pandemic-induced learning environments affected students during COVID-19 and how the influence of interactions with nature impacted DASA-related outcomes. Nonetheless, the study had several limitations. A key limitation in the overall study design was the usage of two different survey tools. This led to an inability to directly compare results from spring/summer to fall. Second, there was a lack of ability to follow individuals over time. Third, female participants represented a large percentage of the participant pool. The university population is approximately 50% female. However, our respondents were disproportionally female (77%). We believe this is likely due to more interest in plants or the incentives given to participants. Female survey participants have been shown to have higher reporting rates for negative emotions over male counterparts [44]. This tendency could have affected the present results. Fourth, a question about race/ ethnicity would have been useful to see if race had any significant effect on the data. The same is true of a question about location of participants. During the Spring/Summer, all students were attending classes online and could have been living anywhere. Different locations could have produced different results, especially when comparing outdoor experiences in urban and rural areas. Furthermore, this could also affect plant access selections due to the number of natural elements that could or could not be present in either rural or urban environments. Another limitation was that we did not inquire why individuals choose to go outside. This might have provided valuable data on the choices being made (e.g., smoke break, humanitarian effort, outdoor exercise) which might have influenced the individuals and also our findings. Researchers adding another response choice in the DASS-based questionnaire holds another limitation since this extra option was not psychometrically validated. Finally, this was written, survey-based research, without an opportunity for follow-up questions and probes to clarify or expand the content of responses. The information given was subject to interpretation by investigators, made through the filter of their personal understanding. 

Despite these limitations, the data from this study present unique findings and suggest some ways in which greenspace and experiences of nature can be designed into students’ surroundings, both in classrooms and throughout campus, to maximize their mental health, academic achievement, and satisfaction with campus life. Future research could enhance these findings by further evaluating the longer-term impacts of the extended pandemic period and considering more variables and how they apply to mental health and nature interactions.

## 5. Conclusions

COVID-19-related stress was found to be a powerful factor that influenced self-reported mental health scores in this study, indicating that the COVID-19 situation itself negatively affected students’ lives. However, plants and nature were found to positively influence students’ mental health and diminish stress to varying degrees. In this study, outdoor experiences positively influenced DASA scores, which showed restorative effects. Furthermore, in the short-term, interactions with plants and nature, both indoors and outdoors, provided some benefits, which was evidenced in reduced depression, anxiety, and stress scores. Nevertheless, this alone does not appear to alleviate the effects of chronic stress and it should be noted that, over time, the benefits of interactions with nature and plants declined.

## Figures and Tables

**Table 1:** Demographics of student respondents to the COVID-19 questionnaire in Spring/Summer and Fall 2020.

Question	Answer	Spring/Summer 2020 *		Fall 2020 *	
		N = 159		N = 194	
Age group	18–24	121	76.1%	153	78.9%
	25–34	31	19.5%	29	15%
	35–44	4	2.5%	8	4.1%
	45–54	3	1.9%	3	1.5%
	55–64	0	-	1	0.5%
	65+	0	-	0	-
	Total:	159	100%	194	100%
Student status	Freshman	15	9.4%	43	22.2%
	Sophomore	25	15.7%	29	15%
	Junior	29	18.2%	33	17%
	Senior	41	25.8%	38	19.6%
	Graduate Student	48	30.2%	50	25.8%
	Not sure	0	-	1	0.5%
	Total:	158	99.4%	194	100%
Gender	Male	30	18.9%	45	23.2%
	Female	123	77.4%	142	73.2%
	Non-binary	4	2.5%	4	2.1%
	Prefer not to say	1	0.6%	3	1.5%
	Total:	158	99.4%	194	100%
Major/Minor involve plants?	Yes	14	8.8%	30	15.5%
	No	144	90.6%	164	84.5%
	Total:	158	99.4%	194	100%
Pre-COVID-19 learning method	On-campus student	145	91.2%	167	86.1%
	Online student	13	8.2%	26	13.4%
	Total:	158	99.4%	193	99.5%

* Totals are for the respondent participation, not for additive questions which resulted in amounts less than 100%. Numbers were rounded to ±1 significant figure for calculation, which led to minor variations in the totals.

**Table 2:** Distribution of students in depression, anxiety, stress, and academic stress rating scales.

		Spring/Summer 2020 *		Fall 2020 *	
	Scale	N = 159		N = 194	
Depression rating	Normal	38	23.90%	38	19.60%
	Mild	17	10.70%	15	7.70%
	Moderate	24	15.10%	33	17%
	Severe	18	11.30%	22	11.30%
	Extremely severe	54	34%	69	35.60%
	Total:	151	95%	177	91.20%
Anxiety rating	Normal	46	28.90%	52	26.80%
	Mild	10	6.30%	11	5.70%
	Moderate	22	13.80%	22	11.30%
	Severe	13	8.20%	11	5.70%
	Extremely severe	60	37.70%	81	41.80%
	Total:	151	95%	177	91.20%
Stress rating	Normal	38	23.90%	46	23.70%
	Mild	14	8.80%	11	5.70%
	Moderate	28	17.60%	21	10.80%
	Severe	16	10.10%	30	15.50%
	Extremely severe	55	34.60%	69	35.60%
	Total:	151	95%	177	91.20%
Academic stress rating	Normal	40	25.20%	33	17%
	Mild	40	25.20%	54	27.80%
	Moderate	29	18.20%	41	21.10%
	Severe	22	13.80%	34	17.50%
	Total:	131	82.40%	162	83.50%

* Totals are for the respondent participation, not for additive questions which resulted in amounts less than 100%. Numbers were rounded to ±1 significant figure for calculation, which led to minor variations in the totals.

**Table 3:** Effects of DASA scores on gender.

	Gender	Spring/Summer	Fall
Depression scale	Female	3.34	3.47
	Male	2.67	3.03
	p depression	0.203	0.227
Anxiety scale ^Z^	Female	3.46 a	3.49
	Male	2.33 b	2.74
	p anxiety	*0.0065*	0.1285
Stress scale ^Z^	Female	3.45 a	3.58 a
	Male	2.5 b	2.71 b
	p stress	*0.0164*	*0.0153*
Academic stress scale	Female	2.3	2.54
	Male	1.91	2.25
	p academic stress	0.279	0.054

^Z^ Different lowercase letters within a column represent significant differences between designated values as specified by Student’s *t*-tests (*p* ≤ 0.05) as appropriate. Italicized *p*-values represent significance at *p* ≤ 0.05. Spring/Summer is missing *n* = 5 and Fall is missing *n* = 7 due to low number of people identifying as non-binary or preferring not to say their gender.

**Table 4:** Influence of going outside on depression, anxiety, stress, and academic stress by semester.

If You Do Go Outside, How Do You Feel When You Return Home?	Spring/ Summer				Fall			
	Depression ^Z^	Anxiety ^Z^	Stress ^Z^	Academic	Depression ^Z^	Anxiety ^Z^	Stress ^Z^	Academic
				Stress ^Z^				Stress ^Z^
I don’t go outside	23.38 b	20.46 ab	33.08 ab	16.67 b	31.23 a	22.67 ab	30.38 ab	23.6 a
I feel worse when I go home	39.86 a	28.29 a	37.29 a	28.73 a	32.23 a	26.71 a	36.71 a	23.47 a
I feel the same when I go home	22.8 b	16.32 b	20.72 d	16 b	21.26 bc	14.32 c	23.11 c	17.94 ab
I feel a little better when I go home	23.05 b	17.16 b	27.61 bc	16.2 b	23.49 b	20.25 ab	29.27 b	20.19 ab
I feel very good when I go home	17.05 bc	11.52 b	21.33 cd	16.5 b	22.58 abc	16.82 bc	25.65 bc	14.63 bc
I feel an enhancement of focus	12.19 c	11.71 b	18.29 d	8.5 c	14.64 c	11.71 c	17.79 c	12.62 c
*p* value	*0.0001*	*0.0041*	*0.0001*	*0.0001*	*0.0011*	*0.0026*	*0.0001*	*0.0029*

^Z^ Different lowercase letters within a column represent significant differences between designated values as specified by Student’s *t*-tests (*p* ≤ 0.05. Italicized *p*-values represent significance at *p* ≤ 0.05.

**Table 5:** Multivariate Correlation Analysis of DASA and survey categories for Spring/Summer. Spearman’s ρ correlation was performed to assess relationships between variables.

	Indoor Plant Exposure	Outdoor Exposure	Workplace Functionality	Workplace Constraints	Difficulties Getting Online	Financial Issues	Plant Access	COVID-19-Related Stress	Beneficial Effects of COVID-19	Depression Rating	Anxiety Rating	Stress Rating	Academic Stress Score
Indoor plant exposure	1												
Outdoor exposure	0.174 *	1											
Workplace functionality	0.104	0.127	1										
Workplace constraints	0.0.147	0.021	−0.164 *	1									
Difficulties getting online	0.093	0.130	0.011	0.363 ***	1								
Financial issues	−0.022	−0.049	−0.077	0.025	0.028	1							
Plant access	0.343 ***	0.236 **	0.195 *	−0.103	0.019	−0.061	1						
COVID-19-related stress	0.014	−0.190 *	−0.084	0.292 ***	0.207 *	0.201 *	−0.051	1					
Beneficial effects of COVID-19	0.060	0.0059	0.019	−0.139	−0.038	−0.007	0.075	−0.212 **	1				
Depression rating	0.103	−0.117	−0.163 *	0.298 **	0.104	0.085	−0.124	0.432 ***	−0.150	1			
Anxiety rating	0.164 *	−0.135	−0.134	0.240 *	0.161	0.085	−0.038	0.380 ***	−0.091	0.748 ***	1		
Stress rating	0.121	−0.181 *	−0.064	0.353 ***	0.179 *	0.082	−0.067	0.455 ***	−0.189	0.790 ***	0.834 ***	1	
Academic stress score	−0.024	−0.088	−0.101	0.244 **	0.135	0.129	−0.093	0.251 **	−0.195 *	0.532 ***	0.534 ***	0.597 ***	1

Values that have *** indicate significance < 0.001. Values that have ** indicate significance < 0.01. Values that have * indicate significance < 0.05.

**Table 6:** Multivariate Correlation Analysis of DASA and survey categories for Fall. Spearman’s ρ correlation was performed to assess relationships between variables.

N = 194	Indoor Plant Exposure	Outdoor Exposure	Workspace Functionality	Workspace Constraints	Difficulties Getting Online	Financial Issues	Plant Access	COVID-19-Related Stress	Beneficial Effects of COVID-19	Depression Rating	Anxiety Rating	Stress Rating	Academic Stress Score
Indoor plant exposure	1												
Outdoor exposure	0.307 ***	1											
Workspace functionality	0.267 **	0.011	1										
Workspace constraints	0.074	0.025	0.006	1									
Difficulties getting online	0.044	0.029	−0.061	0.378 ***	1								
Financial issues	−0.046	0.076	−0.067	0.162 **	0.159 *	1							
Plant access	0.332 ***	0.079	0.316 ***	−0.057	−0.002	−0.082	1						
COVID-19-related stress	0.005	−0.021	−0.017	0.415 ***	0.341 ***	0.196 **	−0.009	1					
Beneficial effects of COVID-19	0.137	0.164 *	0.169 *	−0.135	0.028	−0.074	0.175 *	−0.289	1				
Depression rating	−0.106	−0.236 **	−0.022	0.179 *	0.040	0.189	−0.004	0.441 ***	−0.173 *	1			
Anxiety rating	−0.076	−0.133	−0.009	0.340 ***	0.124	0.189 *	0.11	0.502 ***	−0.228 **	0.645 ***	1		
Stress rating	−0.023	−0.170 *	−0.011	0.30 ***	0.135	0.049	0.057	0.496 ***	−0.253 **	0.641 ***	0.785 ***	1	
Academic stress score	−0.044	−0.248 ***	0.009	0.283 ***	0.191 *	0.188 *	0.036	0.407 ***	−0.241 *	0.583 ***	0.522 ***	0.590 ***	1

Values that have *** indicate significance < 0.001. Values that have ** indicate significance < 0.01. Values that have * indicate significance < 0.05.

## Data Availability

Data are not available upon request because of confidentiality and assurances made to participants.

## Appendix A

**Table A1:** DASA scoring rubric for student surveys.

Meaning	Depression *	Anxiety *	Stress *	Academic Stress
Normal	0–9	0–7	0–14	0–9
Mild	10–13	8–9	15–18	10–19
Moderate	14–20	10–14	19–25	20–29
Severe	21–27	15–19	26–33	30–40
Extremely Severe	28+	20+	34+	-

Based off DASS 21 scale *.

**Table A2:** Workplace functionality rubric for survey answers. Participants were told to select all that applied, and scores were cumulative based on their selections.

Workplace Functionality Scoring Rubric	
Desk	2
Separate room	1
No defined space	−1
Window	1
Next to indoor plants	1
Outside	1
On campus	1

**Table A3:** Workplace constraints rubric for survey answers. Participants were told to select all that applied, and scores were cumulative based on their selections.

Workplace Constraints Scoring Rubric	
Loud	1
Cluttered	1
Poor lighting	1
Sleeping roommate	1
Small work area	1
Children	1
Time	1
Bandwidth	1

**Table A4:** Difficulties getting online assessment rubric for survey answers. Participants were told to select all that applied, and scores were cumulative based on their selections.

Difficulties Getting Online Scoring Rubric	
Equipment	1
Getting online	1
Accessing video/meetings	1
Accessing blackboard	1
Accessing posted materials or lectures	1
Other remote access issues	1
Everything running smoothly	0

**Table A5:** Financial issues assessment for survey answers. Participants were told to select all that applied, and scores were cumulative based on their selections.

Financial Issues due to COVID-19 Scoring Rubric	
I lost my job	1
A family member lost their job	1
My partner lost job	1
No one lost their job	0
Other	0

**Table A6:** Plant access assessment for survey answers. Participants were told to select all that applied, and scores were cumulative based on their selections.

Plant Access Inside Home Working Environment Scoring Rubric	
Window views	1
Real plants	1
Fake pants	1
Work outside	1
Other	0

**Table A7:** COVID-19-related stress assessment for surveys. Participants were told to select all that applied, and scores were cumulative based on their selections.

COVID-19related Stress Scoring Rubric	
No, it has made life easier	0
Yes, this outbreak has caused financial stress on me.	1
Yes, this outbreak has affected my relationships with people.	1
Yes, this outbreak has caused me stress due to being at home.	1
Yes, this outbreak has caused me an increase amount of academic stress.	1
Yes, this outbreak has caused me stress for a reason not listed.	1

**Table A8:** Beneficial views of COVID-19 assessment based on survey answers. Participants were told to select all that applied, and scores were cumulative based on their selections.

Beneficial Viewpoints of COVID-19 as a Student Scoring Rubric	
No, this event is stressful on me as a student (See above).	0
Yes, the recent shift to online school has made school easier.	1
Yes, working at home alleviated commutes to campus making life easier.	1
Yes, the shift made my classes less intense.	1
Yes, the shift allowed me to gain more focus at home.	1
Yes, but for reasons not listed.	1

**Table A9:** Indoor plant exposure assessment for survey answers. Participants were told to select all that applied, and scores were cumulative based on their selections.

Indoor Plant Exposure Scoring Rubric	
No plants	0
Few plants (1–3)	1
Moderate (4–10)	2
Many plants (11+)	3

**Table A10:** Outdoor exposure assessment for survey answers. Participants were told to select all that applied, and scores were cumulative based on their selections.

Outdoor Exposure Scoring Rubric	
Does not take care of plants outside/No job outside/<2 h outside a week	0
Takes care of plants outside	1
Job outside	1
3–6 h outside a week	1
7–12 h outside a week	2
13–20+ h outside a week	3
